# Transient Dysregulation of Dopamine Signaling in a Developing *Drosophila* Arousal Circuit Permanently Impairs Behavioral Responsiveness in Adults

**DOI:** 10.3389/fpsyt.2017.00022

**Published:** 2017-02-13

**Authors:** Lachlan Ferguson, Alice Petty, Chelsie Rohrscheib, Michael Troup, Leonie Kirszenblat, Darryl W. Eyles, Bruno van Swinderen

**Affiliations:** ^1^Queensland Brain Institute, The University of Queensland, Brisbane, QLD, Australia; ^2^Queensland Centre for Mental Health Research, Wacol, QLD, Australia

**Keywords:** schizophrenia, ontogeny, sleep, visual, D1 receptor, genetics

## Abstract

The dopamine ontogeny hypothesis for schizophrenia proposes that transient dysregulation of the dopaminergic system during brain development increases the likelihood of this disorder in adulthood. To test this hypothesis in a high-throughput animal model, we have transiently manipulated dopamine signaling in the developing fruit fly *Drosophila melanogaster* and examined behavioral responsiveness in adult flies. We found that either a transient increase of dopamine neuron activity or a transient decrease of dopamine receptor expression during fly brain development permanently impairs behavioral responsiveness in adults. A screen for impaired responsiveness revealed sleep-promoting neurons in the central brain as likely postsynaptic dopamine targets modulating these behavioral effects. Transient dopamine receptor knockdown during development in a restricted set of ~20 sleep-promoting neurons recapitulated the dopamine ontogeny phenotype, by permanently reducing responsiveness in adult animals. This suggests that disorders involving impaired behavioral responsiveness might result from defective ontogeny of sleep/wake circuits.

## Introduction

The positive symptoms within schizophrenia can be considered as a disorder involving the misattribution of salience (1), where patients tend to respond in a maladaptive way to both external and internally generated stimuli. Salience is largely regulated by dopaminergic systems, and several cognitive disorders involve impaired dopamine signaling (2). The mechanism/s by which an early alteration in dopamine systems might influence aberrant salience allocation in adulthood remains unknown. One hypothesis regarding the etiology of schizophrenia suggests that adverse events *in utero* may alter how dopamine neurons develop, leading to persistent alterations in their function in the adult brain (3, 4). In thinking about what adverse mechanisms may be at play, one possible explanation may be that a transient increase in dopamine signaling during development results in a change in downstream neural machinery, such as arousal-related networks in the brain (5, 6). This may include compensatory changes to postsynaptic dopamine receptors in, for instance the basal ganglia, which is critical in modulating attention *via* dopaminergic circuits (7).

Genetic models such as the fruit fly *Drosophila melanogaster* offer the potential to test the dopamine ontogeny hypothesis in a precisely controlled context (8). *Drosophila* provides several advantages for modeling psychiatric disorders potentially linked to dopaminergic dysregulation. As with humans, dopamine also modulates arousal and attention in *Drosophila* (9, 10), suggesting that similar mechanisms might be involved in allocating salience to stimuli. Since a key aspect of the dopamine ontogeny hypothesis posits a transient effect on dopaminergic signaling during development, it is necessary to develop models that might accurately mimic such temporary changes in dopamine activity or receptor function. Such approaches are readily available using *Drosophila* thermogenetic techniques, by for example controlling receptor expression or channel function with genetically linked temperature shifts (11). Also, high-throughput behavioral paradigms have been developed for *Drosophila* for probing behavioral responsiveness and arousal, and these are being increasingly applied to model cognitive disorders in flies (12).

In a previous study, we found that a transient increase of dopamine activity during fly development permanently altered behavior and brain activity in adult animals (13). Here, we extended that study to examine how this same manipulation affects behavioral responsiveness across two different sensory modalities, mechanosensation and vision in adult animals. We then examined whether a transient reduction in dopamine receptors during development may also recreate these behaviors in adult animals. Finally, we conducted a behavioral screen of various candidate brain circuits to uncover neurons potentially targeted by dopamine to cause these permanent defects in behavioral responsiveness. We then used a thermogenetic strategy to transiently knock down dopamine receptor expression in a subset of these neurons, to determine the likely circuits governing these ontogenetic effects.

## Materials and Methods

### *Drosophila melanogaster* Stocks and Rearing Conditions

Flies were raised at 19°C, 50–60% humidity, 12 h:12 h light:dark cycle on standard yeast-based media. All strains were originally attained from Bloomington *Drosophila* Stock Centre or referenced otherwise. C5-Gal4, 104y-Gal4, GR23E10-Gal4, and GR55B01 were obtained from Paul Shaw (Washington University). Dopamine receptor UAS-RNAi lines (obtained from the VDRC stock center) were used to inhibit gene expression by RNA interference. Both UAS-KK107058 and UAS-KK105834 constructs—inserted on the third chromosome—inhibited synthesis of Dop1R1 and Dop1R2 receptors, respectively. D2 RNAi line was obtained from Scott Waddell (Oxford University). An additional Dicer-Gal4 insertion on the second chromosome was used for all crosses to further promote the action of RNAi by activating the RNA-induced silencing complex and enhancing double-strand RNA cleavage.

### Developmental Intervention and Rearing Conditions

Groups of approximately 30–40 male flies were crossed to 30–40 virgin females. Crosses were allowed 2 days to lay eggs on a standard fly media at 19°C. At the late stage of pupation, when progeny reached 9.5 days old, flies were transferred to a 31°C incubator for 2.5 days. After this, pupae were returned to 19°C until eclosion and behavioral testing as adults (see below). To ensure a developmentally specific effect, post-eclosion, flies were collected within 24 h of heat treatment and transferred into bottles with fresh media. Visual experiments were conducted on 4- to 5-day-old flies, while *Drosophila* ARousal Tracking (DART) was conducted from days 3 to 6 of adulthood. Flies were maintained at 23°C during DART experiments. Temporally controlled expression of dopamine receptors was achieved *via* the inhibitory action of a temperature-sensitive tubulin-Gal80. Temperature protocols for dopamine receptor knockdown experiments were identical to previously published protocols for TrpA1 activation experiments (13).

### Visual Behavior

Flies were collected post-eclosion and wings were excised under CO_2_ anesthesia. After 3 days recovery, flies were tested in the visual arena. Individual flies were transferred to a plastic center platform (86 mm diameter) surrounded by a water moat. The arena was uniformly lit with blue light at a luminance intensity of 770 lx from six surrounding LED panels (128 × 32 individual LED units each, Unity Opto Technology Co., Taiwan). Vision Egg software (14) was used to create the visual stimuli in Python programming language. Visual stimuli were controlled on LEDs with LED Studio Software (Shenzen Sinorad, Medical Electronics, Shenzen, China), at a refresh rate of 200 Hz to ensure background flicker could not be detected by the flies. The visual motion stimulus delivered was a grating rotating around the fly at 3 Hz (speed of 54°/s, with bars of 9° width and 45° height from the center of the arena) and was controlled by LED Studio software (Shenzen Sinorad, Medical Electronics, Shenzen, China). Activity in response to the visual stimuli was filmed (SONY CCD-IRIS video camera) from directly above the arena and tracked using Buritracker software (15). Flies were exposed to 1.5 min of clockwise rotation followed by 1.5 min of counterclockwise rotation, to determine an average optomotor response (the angular velocity of the flies calculated as turning angle per second in the direction of the motion stimulus) as well as locomotion metrics (15). Flies were also exposed to two static bars flickering at 3 Hz, with fixation determined as the smallest angle of deviation between the fly’s trajectory and either of the two vertical bars (15). These two visual paradigms (optomotor and fixation) measure two different forms of visual responsiveness in flies, namely motion perception and object perception. Optomotor behavior is indicated by a high optomotor index, whereas fixation is indicated by a low angle of deviation, α (15).

### Sleep and Arousal

Sleep and behavioral responsiveness were determined by using the DART platform as described previously (16). Briefly, 2- to 3-day-old male flies were collected under CO_2_ anesthesia, transferred individually into 65 mm glass tubes (Trikinetics, Waltham, MA), and then maintained at 23°C on a 12 h:12 h light:dark cycle. Seventeen tubes on each plastic tray with three to six trays per experiment were filmed continuously for 72 h using a USB-webcam (Logitech) fitted with a wide-angle lens (Zeiss). DART software controlled the delivery of vibrational stimuli once every hour. Shaft-less vibrating motors (Precision Microdrives™; model 312–101) were glued underneath each tray (two motors per tray) and an input voltage of 3.5 V delivered 2.4 g vibration amplitude. The DART platform provided a range of metrics used in this study, including sleep duration and intensity, and statistical analyses were performed within the DART analysis suite.

### High-Performance Liquid Chromatography

Quantification of DA levels in fly heads was performed using high-performance liquid chromatography (HPLC). The system consisted of an autosampler and an isocratic pump (Model 1100, Agilent Technologies, Inc., CA, USA), coupled to a Sunfire C18 column (4.6 mm × 150 mm, 5 μm; Waters Corporation, MA, USA), and a Coulochem III electrochemical detector (ESA Laboratories, Inc., MA, USA). The working electrode was set to a potential of +300 mV. The mobile phase consisted of 25 mM sodium dihydrogen phosphate anhydrous, 50 mM citric acid monohydrate, 1.4 mM octane sulfonic acid, and 1 mM EDTA. The pH was adjusted to 4.22, and 6% acetonitrile was added. Neurotransmitter standards were prepared daily using 0.1 M perchloric acid. The internal standard solution was deoxyepinephrine (DE) at 250 ng/mL. Between 5 and 10 fly heads were pooled for each genotype. Heads were dissected freehand and placed directly into a solution of 100 μL perchloric acid and 20 μL DE. The heads were homogenized then centrifuged and 25 μL injected. Samples were analyzed in duplicate. Identification and quantification of neurotransmitter peaks was performed using ChemStation software. Results were displayed as picograms of DA per head (pg/head).

### Gene Expression

A quantitative reverse transcriptase PCR assay was used to confirm knockdown of the Dop1R1 and Dop1R2 receptor genes relative to that of the housekeeping gene Act88F (17). Act88F was first determined to be stably expressed across all experimental conditions (data not shown). Flies were collected by CO_2_ anesthesia as either pupae or adults, snap frozen, and stored at −80°C. Six pools of five fly heads (30 heads total) were placed into a 1.5-mL Eppendorf tube. Total RNA was purified using TRIzol according to the manufacturer’s protocols (Invitrogen, Carlsbad, CA, USA), immediately after dissection. Total RNA was treated with DNase (Sigma-Aldrich, St. Louis, MO, USA) to eliminate genomic DNA. Approximately 0.5 μg of total RNA was reverse transcribed using random primers (Invitrogen) and reverse transcriptase (Invitrogen) according to the manufacturer’s protocols. Gene expression was estimated with two technical replicates using a standard quantitative PCR (qPCR) assay (17). Each qPCR mixture contained 12.5 μL of 2X SYBR premix (Invitrogen), 1 μL of forward primer, 1 μL of reverse primer, 100 ng of DNA, and H2O to a final volume of 25 μL. The expression of the two genes was estimated relative to Act88F using the CT (where CT is threshold cycle) method (18). Averages of expression were compared using Student’s *t*-test (SPSS). Data were log-transformed as per established methods for analyzing gene expression differences (19).

### Statistical Analysis

Behavioral data were analyzed for statistical significance using SPSS and Prism software (GraphPad Software, La Jolla, CA, USA). Sleep and arousal behavioral data were examined using one-way analysis of variance (ANOVA, α < 0.05) to compare between grouped means. Visual behavioral data were examined using multivariate analysis of variance (MANOVA, α < 0.05) to compare grouped means of individual flies per genotype. RNA knockdown effects were determined by ANOVA. Sleep and arousal effects for the genetic screen were determined by *t*-test. Where significance occurred between group means, *post hoc* Tukey’s multiple comparison tests (*P* < 0.05) were used to determine significant differences between transgenic mutants and genetic controls for these experiments.

## Results

### Transient Elevation in Presynaptic Dopamine: Effects on Adult Responsiveness

**Figure 1:**
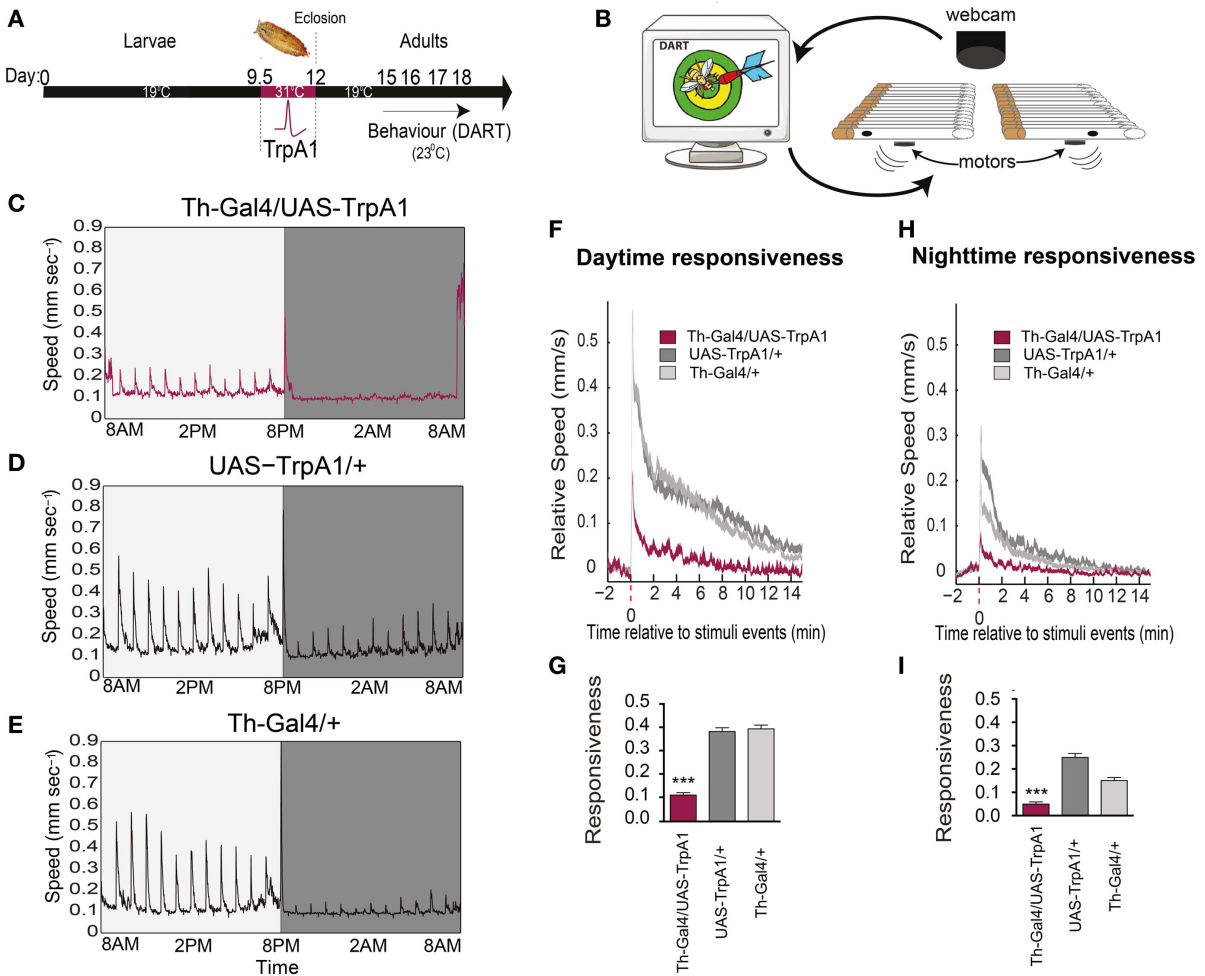
**Transient activation of dopamine during development decreases behavioral responsiveness to mechanical stimuli in adults**. **(A)** Timeline of experiment. Th-Gal4/UAS-TrpA1 flies were exposed to elevated temperatures (31°C) during their late pupal stage, which activates dopaminergic neurons specifically. Behavioral experiments were then performed on adult males at room temperature, using the *Drosophila* ARousal Tracking (DART) system. **(B)** Adult flies were placed in individual tubes with access to food, and their responsiveness to mechanical stimuli (vibrating motors) was monitored hourly over 3 days and nights using DART. **(C)** Average speed (mm/s) of Th-Gal4/UAS-TrpA1 flies (*N* = 32) to hourly mechanical vibrations for day (light gray) and night (dark gray). **(D)** Average speed of identically treated UAS-TrpA1/+ genetic controls (*N* = 32). **(E)** Average speed of identically treated Th-Gal4/+ genetic controls (*N* = 32). **(F)** Average daytime responsiveness of treated Th-Gal4/UAS-TrpA1 animals (maroon) compared to genetic controls (gray). **(G)** Average responses are compared to each other by zeroing the baseline (pre-stimulus) speed, and summarized average daytime responsiveness (mm/s ± SEM) is shown in the histogram. **(H)** Average nighttime responsiveness of treated Th-Gal4/UAS-Gal4 animals (maroon) compared to genetic controls (gray). **(I)** Average nighttime responsiveness (mm/s ± SEM) for the three strains. ***P < 0.001, by one-way ANOVA, adjusted for multiple comparisons by *Post Hoc* Tukey’s test.

**Figure 2:**
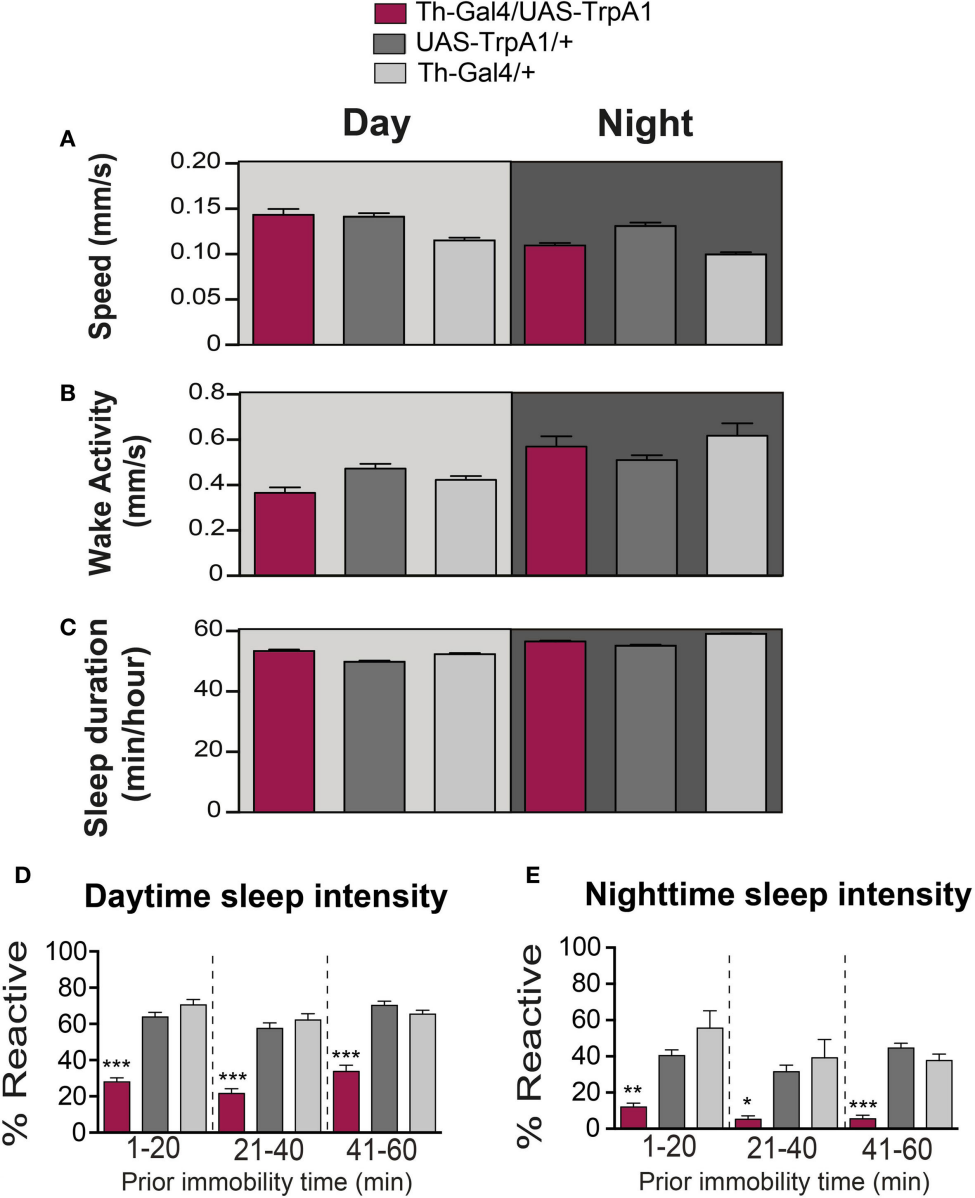
**Transient activation of dopamine during pupal development does not alter waking activity or sleep duration**. **(A)** Average daytime (light-gray background) and nighttime (dark-gray background) pre-stimulus speed (mm/s ± SEM) for treated Th-Gal4/UAS-TrpA1 animals (maroon, *N* = 32) compared to UAS-TrpA1/+ (dark gray, *N* = 32) and Th-Gal4/+ (light gray, *N* = 32). Flies are the same as in Figure 1. **(B)** Average daytime (light-gray background) and nighttime (dark-gray background) wake activity (mm/s speed per waking minute ± SEM, see Materials and Methods) for treated Th-Gal4/UAS-TrpA1 animals (maroon) compared to genetic controls (gray). **(C)** Average daytime (light-gray background) and nighttime (dark-gray background) sleep duration in minutes of sleep/hour ± SEM for treated Th-Gal4/UAS-TrpA1 animals (maroon) compared to genetic controls (gray). **(D)** Average daytime sleep intensity (% immobile flies that reacted to the stimulus ± SEM). Data [same color scheme as in **(A–C)**] are divided into three groups, depending on how long flies were immobile prior to the stimulus event. **(E)** Average nighttime sleep intensity (% reactive ± SEM). *P < 0.05, **P < 0.01; ***P < 0.001, by one-way ANOVA, adjusted for multiple comparisons by *Post Hoc* Tukey’s test.

We investigated whether a transient developmental increase in dopamine activity during *Drosophila* development (Figure 1A) affected behavioral responsiveness in adults, by using a newly developed paradigm to measure behavioral responsiveness to mechanical stimuli in flies, the DART system (16) (Figure 1B). We utilized a heat-inducible genetic construct [TrpA1 (20)] to induce depolarization in dopaminergic neurons during late pupation (21, 22). We found that this transient increase of presynaptic dopamine activity resulted in decreased responsiveness to mechanical stimuli in these flies as adults (Figure 1C), compared to similarly treated genetic controls (Figures 1D,E). This effect was evident as significantly decreased average speed of the treated flies in response to mechanical stimuli during the day (*F*_(2,93)_ = 119, *P* < 0.001, Tukey’s) (Figures 1F,G) and night (*F*_(2, 93)_ = 52.43, *P* < 0.001, Tukey’s) (Figures 1H,I), suggesting a general deficit in arousal. Average walking speed, however, was not significantly affected by this treatment (day: *F*_(2, 93)_ = 11.76, *P* = 0.9494, Tukey’s; night: *F*_(2, 93)_ = 27.53, *P* = 0.0689, Tukey’s) (Figure 2A), suggesting that the deficit is more specifically related to behavioral responsiveness than baseline activity.

As well as being critical for behavioral responsiveness, dopamine is a key regulator of sleep/wake cycles in flies (9, 10, 23–25). We therefore questioned whether the transient manipulation of presynaptic dopamine activity during development would have produced persistent effects on sleep/wake behavior in adult animals. Sleep behavior in *Drosophila* has traditionally been measured by the cumulative duration of quiescence bouts longer than 5 min (26, 27). Treated flies were not less active than controls while awake (*F*_(2, 93)_ = 6.847, *P* = 0.2331, Tukey’s) (Figure 2B). Average sleep duration of the developmentally manipulated flies was not significantly affected for daytime sleep (*F*_(2, 93)_ = 17.03, *P* = 0.2331, Tukey’s) (Figure 2C). Average sleep duration during the night did not show a consistent change compared to both controls but was significantly less (*F*_(2, 93)_ = 6.847, *P* = < 0.001, Tukey’s) than Th-Gal4/+ control flies and significantly more (*F*_(2, 93)_ = 6.847, *P* < 0.01, Tukey’s) than the UAS-TrpA1/+ control flies (Figure 2C).

Another way of measuring sleep is by probing for behavioral responsiveness only in quiescent animals, which can provide insight into sleep intensity (16, 28). To better visualize sleep intensity at different sleep times, behavioral responsiveness (% of flies reacting to the stimulus) was partitioned into three successive prior immobility epochs, 1–20, 21–40, and 41–60 min (Figures 2D,E). Interestingly, our developmental manipulation led to increased sleep intensity in quiescent adults, for both daytime (*F*_(8, 279)_ = 45.94; 1–20 min: *P* < 0.001; 21–40 min: *P* < 0.001; 41–60 min: *P* < 0.001, Tukey’s) and nighttime sleep (*F*_(8, 279)_ = 11.97; 1–20 min: *P* < 0.01; 21–40 min: *P* < 0.05; 41–60 min: *P* < 0.001, Tukey’s) (Figures 2D,E). Thus, treated flies are not sleeping more (or less); they are sleeping more deeply. This finding confirms that arousal is impaired in developmentally manipulated flies and that dysregulation of dopamine during development permanently affects behavioral responsiveness in general, regardless of whether animals are awake or asleep.

### Transient Elevation in Presynaptic Dopamine: Effects on Adult Visual Fixation

**Figure 3:**
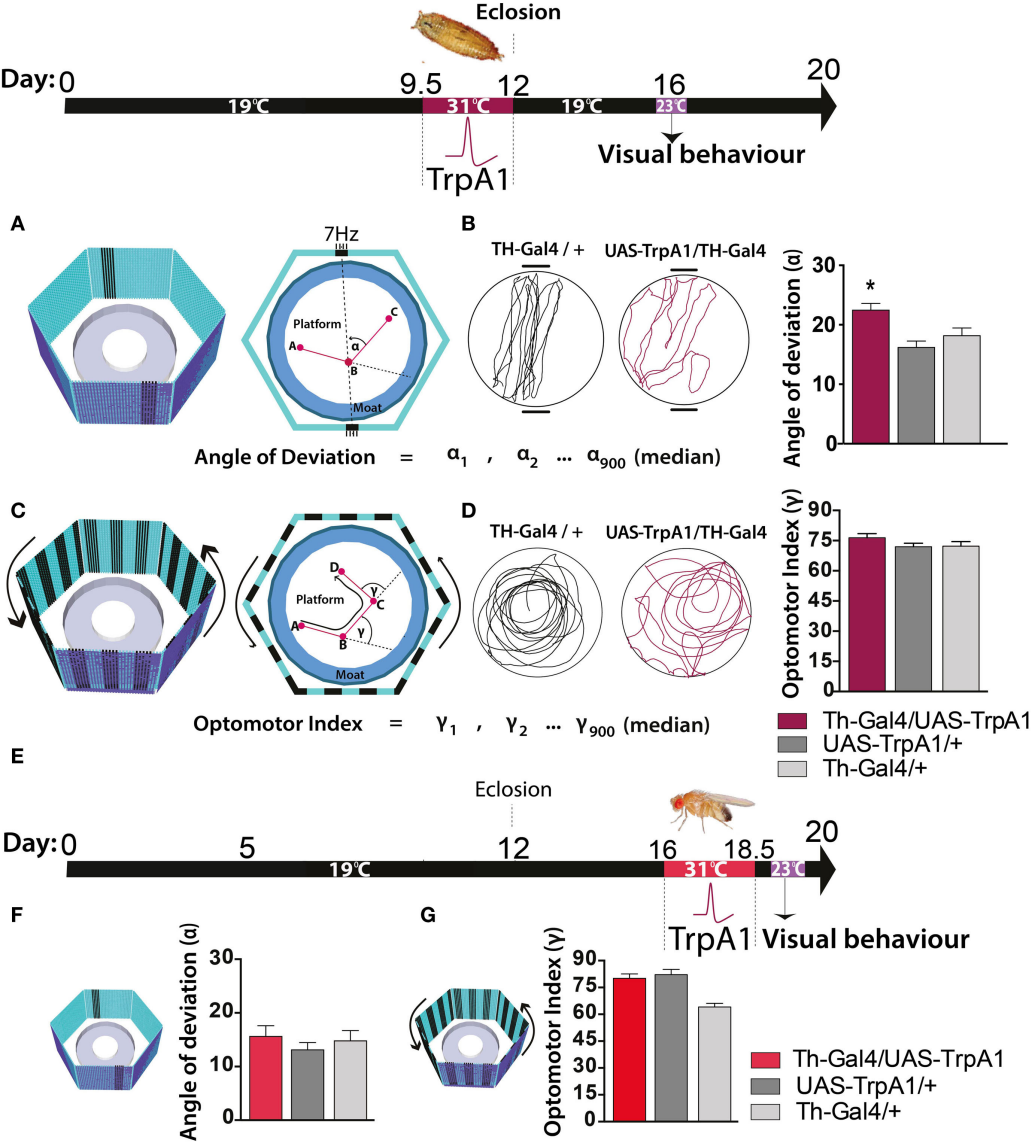
**Transient activation of dopamine during pupal development decreases visual responsiveness in adults**. Top: timeline of experiment, exactly the same as in Figure 1. **(A)** Left panel: the visual arena, consisting of a circular platform surrounded by a moat of water surrounded by six LED arrays displaying virtual objects, two dark bars on a blue background. Right panel: fixation directed to the vertical bars is calculated as the median angle of deviation {(α_900_ + 1) ÷ 2}^th^ value (see Materials and Methods). **(B)** Left panel: example of a fixation trace for a typical treated genetic control (Th-Gal4/+, black) and a typical treated Th-Gal4/UAS-TrpA1 fly (maroon). Right panel: average angle of deviation (±SEM) for Th-Ga4/UAS-TrpA1 (maroon, *N* = 30), UAS-TrpA1/+ (light gray, *N* = 30), and for Th-Gal4/+ (dark gray, *N* = 28). *P < 0.05, by MANOVA between grouped means, adjusted for multiple comparisons by a *Post Hoc* Tukeys test. **(C)** Left panel: the same visual arena as in **(A)**, but with a moving grating displayed on the LEDs. Right panel: optomotor responsiveness is calculated as median optomotor index (OI) = {(γ_900_ + 1) ÷ 2}^th^ value (see Materials and Methods) **(D)** Left panel: example optomotor trace for a treated genetic control (Th-Gal4/+, black) and a treated Th-Gal4/UAS-TrpA1 fly (maroon). Right panel: average OI (±SEM) for Th-Ga4/UAS-TrpA1 (maroon, *N* = 18), UAS-TrpA1/+ (light gray, *N* = 20), and for Th-Gal4/+ (dark gray, *N* = 18). **(E)** Timeline of experiment where dopamine neurons are transiently activated in adult flies. **(F)** Average angle deviation (±SEM) of adult flies treated as in E for Th-Ga4/UAS-TrpA1 (red, *N* = 10), UAS-TrpA1/+ (light gray, *N* = 10), and for Th-Gal4/+ (dark gray, *N* = 10). **(G)** OI (±SEM) of adult flies treated as in **(E)** for Th-Ga4/UAS-TrpA1 (red, *N* = 44), UAS-TrpA1/+ (light gray, *N* = 48), and for Th-Gal4/+ (dark gray, *N* = 50).

We next investigated whether these persistent effects on behavioral responsiveness generalized to other sensory modalities, such as vision. To test visual responsiveness in *Drosophila*, we used a modified version of “Buridan’s paradigm” (29). In this assay, individual flies display visual responsiveness (or “fixation strength”) by walking back and forth between two opposing salient target objects (Figure 3A, left panel). Fixation strength is measured by the angle of deviation to the targets, where a smaller deviation indicates stronger fixation (Figure 3A, right panel). We found that the transient increase in dopamine activity during development significantly impaired fixation to this visual stimulus, as evident by an increased average angle of deviation from the target objects (Figure 3B, maroon), compared to similarly treated controls (*F*_(2,85)_ = 7.77, *P* < 0.05, Tukey’s) (Figure 3B, gray). We questioned whether other forms of visual responsiveness might also be compromised. Specifically, we examined the optomotor response, whereby flies reflexively track moving objects (30) (Figure 3C). We found no significant effect on optomotor behavior in this paradigm (*F*_(2,119)_ = 1.3, *P* = 0.34, Tukey’s) (Figure 3D). Importantly, there were no persistent behavioral effects in either paradigm when dopamine activity was transiently increased in adulthood (fixation paradigm: *F*_(8,46)_ = 2.5, *P* = 0.90, Tukey’s; optomotor paradigm: *F*_(8,270)_ = 5.4 *P* = 0.64, Tukey’s) (Figures 3E–G, red bars).

**Figure 4:**
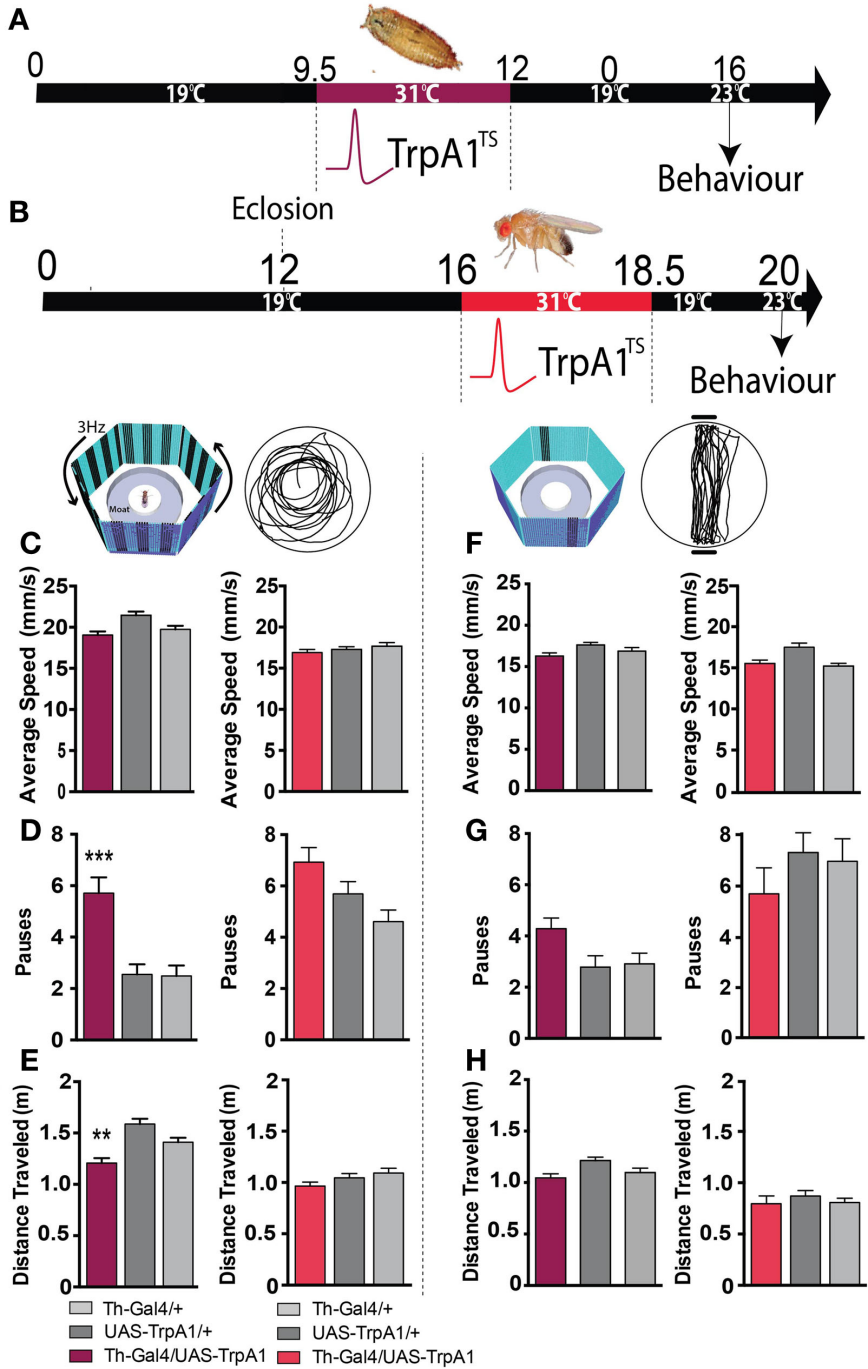
**Transient activation of dopamine during development induces hypolocomotion in adults only under specific visual conditions**. **(A)** Timeline of experiment, exactly the same as in Figure 1. **(B)** Timeline of experiment where dopamine neurons are transiently activated in adult flies. **(C)** Average speed (mm/s ± SEM) for developmentally treated Th-Ga4/UAS-TrpA1 (left panel, maroon) and adulthood-treated Th-Ga4/UAS-TrpA1 (right panel, red) compared to genetic controls (gray) in response to rotating stimuli. **(D)** Number of pauses (±SEM) for developmentally treated Th-Ga4/UAS-TrpA1 (maroon) and adulthood-treated Th-Ga4/UAS-TrpA1 (red) compared to genetic controls (gray) in response to rotating stimuli. **(E)** Total distance traveled (m ± SEM) for developmentally treated Th-Ga4/UAS-TrpA1 (maroon) and adulthood-treated Th-Ga4/UAS-TrpA1 (red) compared to genetic controls (gray) in response to rotating stimuli. **(F)** Average speed (mm/s ± SEM) for developmentally treated Th-Ga4/UAS-TrpA1 (left panel, maroon) and adulthood-treated Th-Ga4/UAS-TrpA1 (right panel, red) compared to genetic controls (gray) in response to stationary objects. **(G)** Number of pauses (±SEM) for developmentally treated Th-Ga4/UAS-TrpA1 (maroon) and adulthood-treated Th-Ga4/UAS-TrpA1 (red) compared to genetic controls (gray) in response to stationary objects. **(H)** Distance traveled (m ± SEM) for developmentally treated Th-Ga4/UAS-TrpA1 (maroon) and adulthood-treated Th-Ga4/UAS-TrpA1 (red) compared to genetic controls (gray) in response to stationary objects. Developmentally treated: Th-Ga4/UAS-TrpA1 (maroon, *N* = 36), UAS-TrpA1/+ (light gray, *N* = 39), and for Th-Gal4/+ (dark gray, *N* = 37). Adult treated: Th-Ga4/UAS-TrpA1 (red, *N* = 10), UAS-TrpA1/+ (light gray, *N* = 10), and for Th-Gal4/+ (dark gray, *N* = 10). **P < 0.01, ***P < 0.001, by MANOVA between grouped means, adjusted for multiple comparisons by a *Post Hoc* Tukey’s test.

Closer examination of individual fly behavior within both these paradigms following manipulation at either the pupal or adult stage revealed that treated flies had a similar average speed as genetic controls (Figures 4A–C,F, maroon and red bars). However, there was a subtle yet significant locomotion defect: flies treated as pupae paused significantly more, but only in the presence of a moving grating designed to evoke an optomotor response (*F*_(2,119)_ = 14.4, *P* < 0.001, Tukey’s) (Figure 4D, maroon). Accordingly, total distance traveled was decreased in these same flies (F_(2,119)_ = 14.9, *P* < 0.01 Tukey’s) (Figure 4E, maroon). Interestingly, none of these locomotion defects were evident when flies were presented with the fixation stimuli [speed (*F*_(2,85)_ = 3.35, *P* = 0.5); pauses (*F*_(2,85)_ = 3.79, *P* = 0.07); distance (*F*_(2,85)_ = 0.48, *P* = 0.636)] (Figures 4F–H, maroon). Taken together, these results suggest two different effects of the developmental manipulation on visual behavior: decreased visual responsiveness when flies are presented with objects to fixate on, and increased pausing (or “freezing”) when flies are presented with moving gratings. Importantly, there were no persistent behavioral effects when dopamine activity was transiently increased only later in adulthood (Figures 3E–G and 4C–H, red bars).

**Figure 5:**
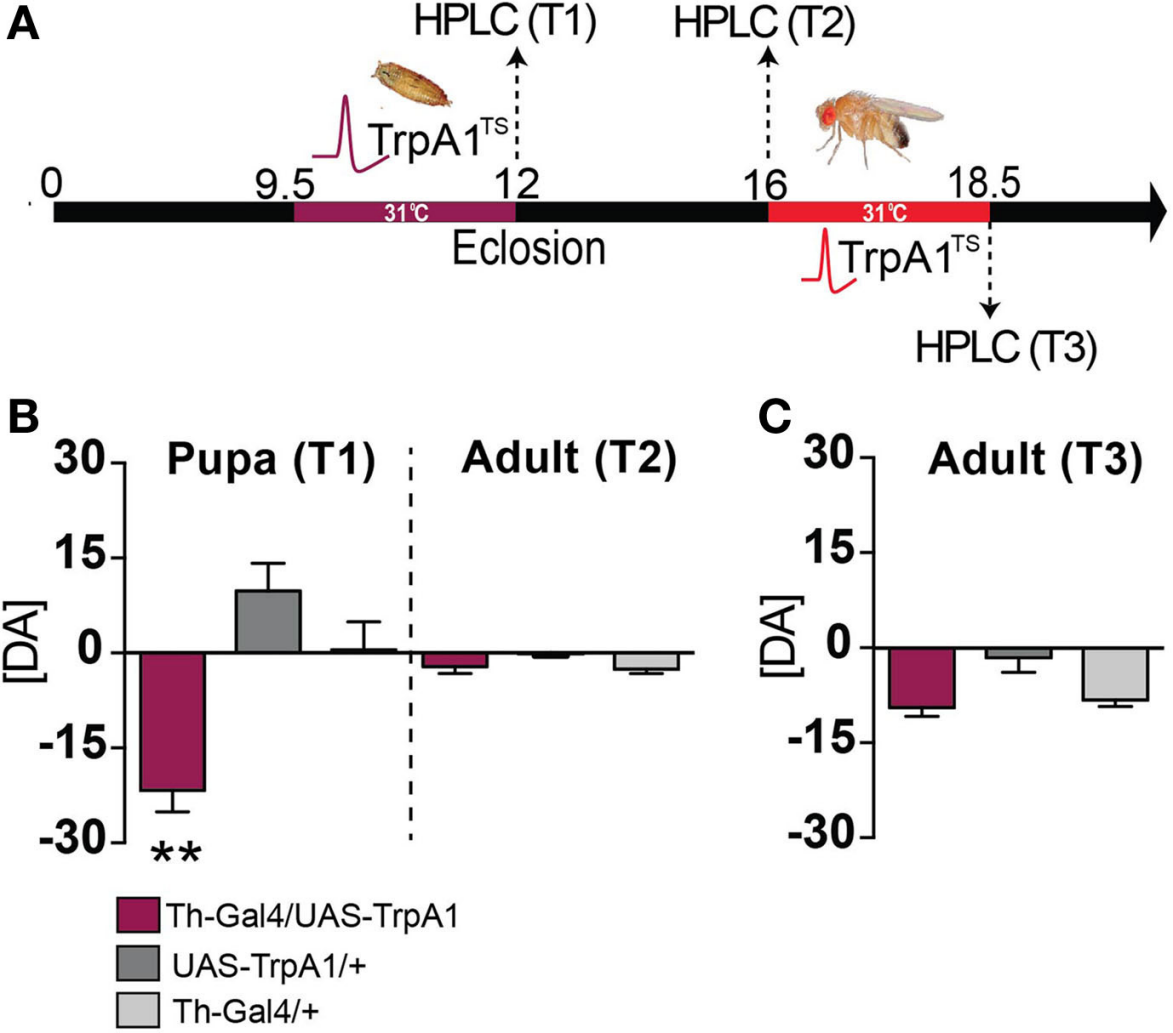
**Dopamine levels in pupae and adults following developmental and adult manipulation of dopamine activity**. **(A)** High-performance liquid chromatography (HPLC) was performed on heads collected from pupae or adults, following a heat treatment in the pupal stage (T1 and T2) or following a heat treatment in the adult stage (T3). **(B)** Developmental manipulation. Left: average pupal dopamine levels ([DA] ± SEM) zeroed to wild type levels [*N* = 7 (35 flies)], for Th-Ga4/UAS-TrpA1 [maroon, *N* = 6 (30 flies)], UAS-TrpA1/+ [dark gray, *N* = 6 (30 flies)] and Th-Gal4/+ [light gray, *N* = 6 (30 flies)]. Right: average adult dopamine levels ([DA] ± SEM) zeroed to wild type [*N* = 9 (45 flies)] for Th-Ga4/UAS-TrpA1 [maroon, *N* = 8 (40 flies)], UAS-TrpA1/+ [gray, *N* = 8 (40 flies)] and Th-Gal4 [*N* = 8 (40 flies)] levels. **P < 0.01 by one-way ANOVA, adjusted for multiple comparisons by a *Post Hoc* Tukey’s test. **(C)** Adult manipulation. Average adult dopamine levels ([DA] ± SEM) zeroed to wild type [*N* = 7 samples (35 flies)] levels, for Th-Ga4/UAS-TrpA1 [maroon, *N* = 7 samples (35 flies)], UAS-TrpA1/+ [gray, *N* = 11 samples (55 flies)] and Th-Gal4/+ [light gray *N* = 6 samples (30 flies)]. Significance tested by one-way ANOVA, adjusted for multiple comparisons by a *Post Hoc* Tukey’s.

We next examined whether a transient increase in dopamine activity during development might have produced long-term effects on dopamine levels in adult animals. Dopamine levels were measured in the heads of pupal flies immediately after treatment, and in adult flies that had undergone the developmental manipulation (see Materials and Methods). We found that dopamine levels were significantly decreased in pupal brains immediately following the manipulation, compared to control pupae (Figures 5A,B, T1) (*F*_(2, 15)_ = 15.48, *P* < 0.01, Tukey’s). Intriguingly, dopamine levels returned to normal by adulthood (Figure 5B, T2) [*F*_(2, 15)_ = 2.691, *P* = 0.1003]. In contrast, transient DA manipulations in adult flies had no effect on DA levels (Figure 5C, T3) (*F*_(2, 17)_ = 6.049, *P* = 0.8814). Our finding, that whole-brain dopamine levels are unchanged in treated animals at the adult stage (when the aberrant behaviors are manifest), combined with previous results indicating that gross dopamine circuitry seems unaffected by this developmental manipulation (13), suggests that the transient increase in dopamine transmission during development may have altered dopaminergic connectivity rather than producing a persistent alteration in DA levels. Given the plastic nature of the synapse, it is likely that an initial increase in pupal DA release (and subsequent decrease in pupal DA levels) may have possibly induced a compensatory reduction in postsynaptic dopamine receptors. Longer-term increase in DA synthesis has been shown to decrease DA receptor levels in *Drosophila* (31), so it is possible that a transient increase in DA activity during development may also affect receptor expression levels.

### Transient Postsynaptic Manipulations Mimic Presynaptic Dopamine Effects

**Figure 6:**
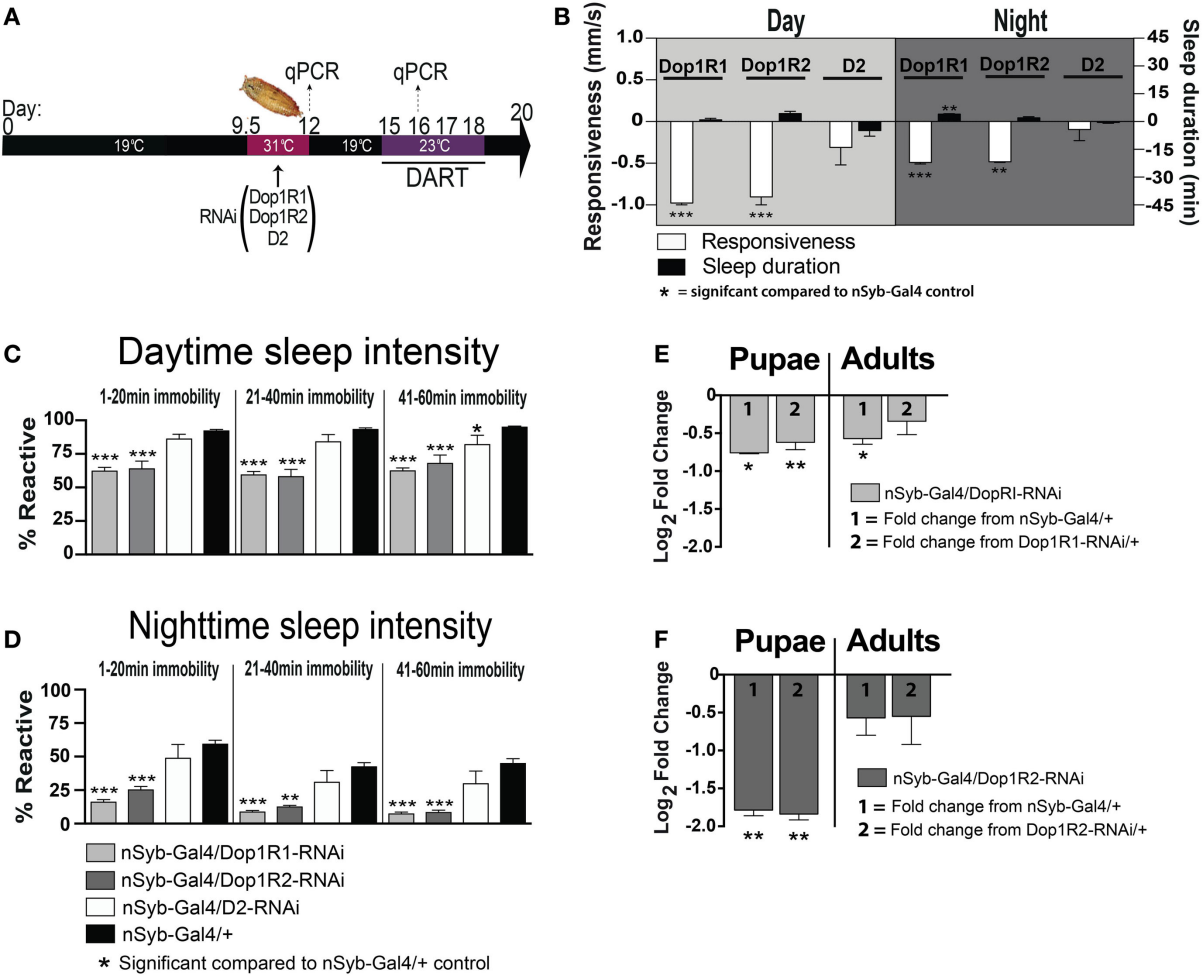
**Transient pan-neuronal knockdown of Dop1R1 and Dop1R2 during development recapitulates arousal defects in adult animals**. **(A)** Timeline of experiment, as in Figure 1, except that the heat treatment produces knockdown of D1 or D2 receptors. **(B)** Average responsiveness (white bars, mm/s ± SEM) or sleep duration (black bars, min ± SEM) during the day and night for treated nSyb-Gal4/UAS-Dop1R1/R2 RNAi or D2 RNAi; tubulin (tub)-Gal80TS animals (*N* = 132, 66, and 75, respectively) compared to nSyb-Gal4/+; tub-Gal80TS genetic controls, set as zero (*N* = 228). **(C)** Daytime sleep intensity (% reactive ± SEM) for the same flies as in **(B)**. **(D)** Nighttime sleep intensity (% reactive ± SEM) for the same flies as in **(B)**. **(E)** Relative gene expression (±SEM) for Dop1R1 in pupae and adults following developmental knockdown. **(F)** Relative gene expression (±SEM) for Dop1R2 in pupae and adults following developmental knockdown. Fold change compared to either genetic control (1 or 2) is shown. ***P < 0.001; **P < 0.01; *P < 0.05, by one-way ANOVA, adjusted for multiple comparisons by a *Post Hoc* Tukey’s test.

Developmental alterations in the dopaminergic environment may affect neural circuitry through postsynaptic changes that outlast the original dopaminergic insult. In healthy humans, it is known that postsynaptic receptor expression can be modulated to compensate for changes in presynaptic activity (32). A transient increase in dopamine activity during development may thus be expected to lead to a downregulation of postsynaptic dopamine receptors. While this adjustment might help maintain normal neurotransmitter activity within an optimal range during the developmental insult, these compensatory postsynaptic effects could be maladaptive once dopamine levels have returned to normal in adulthood (as suggested by our HPLC data in Figure 5). To test this hypothesis, we used RNAi constructs to transiently knock down two different dopamine receptor types (D1 and D2) across the *Drosophila* brain. This was performed during the same critical pupal window of brain development as described above. Pan-neuronal knockdown of these receptors was achieved by placing nSyb-Gal4; Gal80TS/UAS-D1 (or D2)-RNAi flies at an elevated temperature (31°C) for 2.5 days (Figure 6A), such that receptor knockdown was only permitted at the elevated temperature. Crucially, this means these flies underwent the same heat induction treatment as those used for the presynaptic manipulation. The only difference is that in these flies, the effect was to transiently down-regulate dopamine receptors across the brain, rather than to increase dopaminergic activity.

We found that transient developmental knockdown of two distinct D1 receptors (Dop1R1 and Dop1R2) in all neurons significantly decreased behavioral responsiveness in adult animals, during the day [*F*_(3, 30)_ = 16.30, Dop1R1 (*P* < 0.001), Dop1R2 (*P* < 0.001), Tukey’s] and night [*F*_(3, 30)_ = 13.12, Dop1R1 (*P* < 0.001), Dop1R2 (*P* < 0.01) Tukey’s] (Figure 6B, white bars). This mimics the effect of transiently increasing presynaptic dopamine activity during development (Figure 1). In contrast, transient knockdown of the D2 receptor had no significant effect on responsiveness during the day (*P* > 0.05, Tukey’s) or night (*P* > 0.05, Tukey’s) (Figure 6B, D2-white bars). Consistent with the effect of D1 knockdown on behavioral responsiveness, sleep intensity was increased in the D1 manipulated animals [day (*F*_(11, 90)_ = 34.35, Dop1R1 and Dop1R2 all sleep epochs, *P* < 0.001, Tukey’s); night (*F*_(11, 90)_ = 18.55; 1–20 min, Dop1R1, *P* < 0.001; Dop1R2, *P* < 0.001; 21–40 min, Dop1R1, *P* < 0.001; Dop1R2, *P* < 0.01; 41–60 min, Dop1R1 and Dop1R2, *P* < 0.001, Tukey’s)] (Figures 6C,D, gray bars). D2 knockdown during development however had no effect on sleep intensity during the night [night (*F*_(11, 90)_ = 18.55, all sleep epochs *P* > 0.05, Tukey’s)], with only a slight effect during the day in the final 41–60 min epoch [day (*F*_(11, 90)_ = 34.35, 1–20 and 21–40 min, *P* > 0.05, Tukey’s; 41–60 min, *P* < 0.05, Tukey’s)] (Figures 6C,D, white bars). Although D2 receptor malfunction has been linked to hypolocomotion in a previous *Drosophila* study (33), a large volume of other work has more specifically linked D1 receptors in the central fly brain to sleep and arousal phenotypes (24, 25, 34–36). We therefore focused on the D1 receptor subtype in subsequent experiments.

We confirmed by RT qPCR that Dop1R1 and Dop1R2 expression was significantly decreased immediately following the transient manipulation in pupae [Dop1R1 Pupae: (*F*_(2,15)_ = 5.98; *P* = 0.012); nSyb-Gal4/+, *P* = 0.015, +/Dop1R1-RNAi, *P* = 0.003; Dop1R2 Pupae: (*F*_(2,15)_ = 3.204; *P* = 0.069); nSyb-Gal4/+, *P* = 0.006, +/Dop1R2-RNAi, *P* = 0.004; ANOVA; Figures 6E,F, pupae]. Interestingly, Dop1R1 expression remained lower into adulthood, although this was significant only compared to one genetic control therefore unlikely to be functionally relevant [Dop1R1 Adult: (*F*_(2,15)_ = 2.75; *P* = 0.095); nSyb-Gal4/+, *P* = 0.022, +/Dop1R1-RNAi, *P* = 0.273; ANOVA; Figure 6E, adults]. Dop1R2 knockdown did not persist into adulthood [Dop1R2 Adult: *F*_(2,15)_ = 0.81; *P* = 0.46; nSyb-Gal4/+, *P* = 0.247, +/Dop1R2-RNAi, *P* = 0.302; ANOVA; Figure 6F, adults]. Our results show that transiently knocking down D1 receptors (either Dop1R1 and Dop1R2) during development mimics the effects of transiently increasing dopamine activity during the same stage of fly development. Consistent with our DA activation effects, D1 knockdown across the fly brain did not greatly alter sleep duration during the day (*F*_(3, 30)_ = 4.252, *P* > 0.05, Tukey’s) or night (*F*_(3, 30)_ = 6.726, Dop1R2, *P* > 0.05, Tukey’s)—however, we noted a small but significant ~4 min increase in nighttime sleep following transient Dop1R1 knockdown (*P* < 0.01, Tukey’s) (Figure 6B, black bars). Thus, global D1 knockdown at the pupal stage significantly decreases behavioral responsiveness, largely without impacting sleep duration.

### Activating Sleep-Promoting Neurons Decreases Behavioral Responsiveness

**Figure 7:**
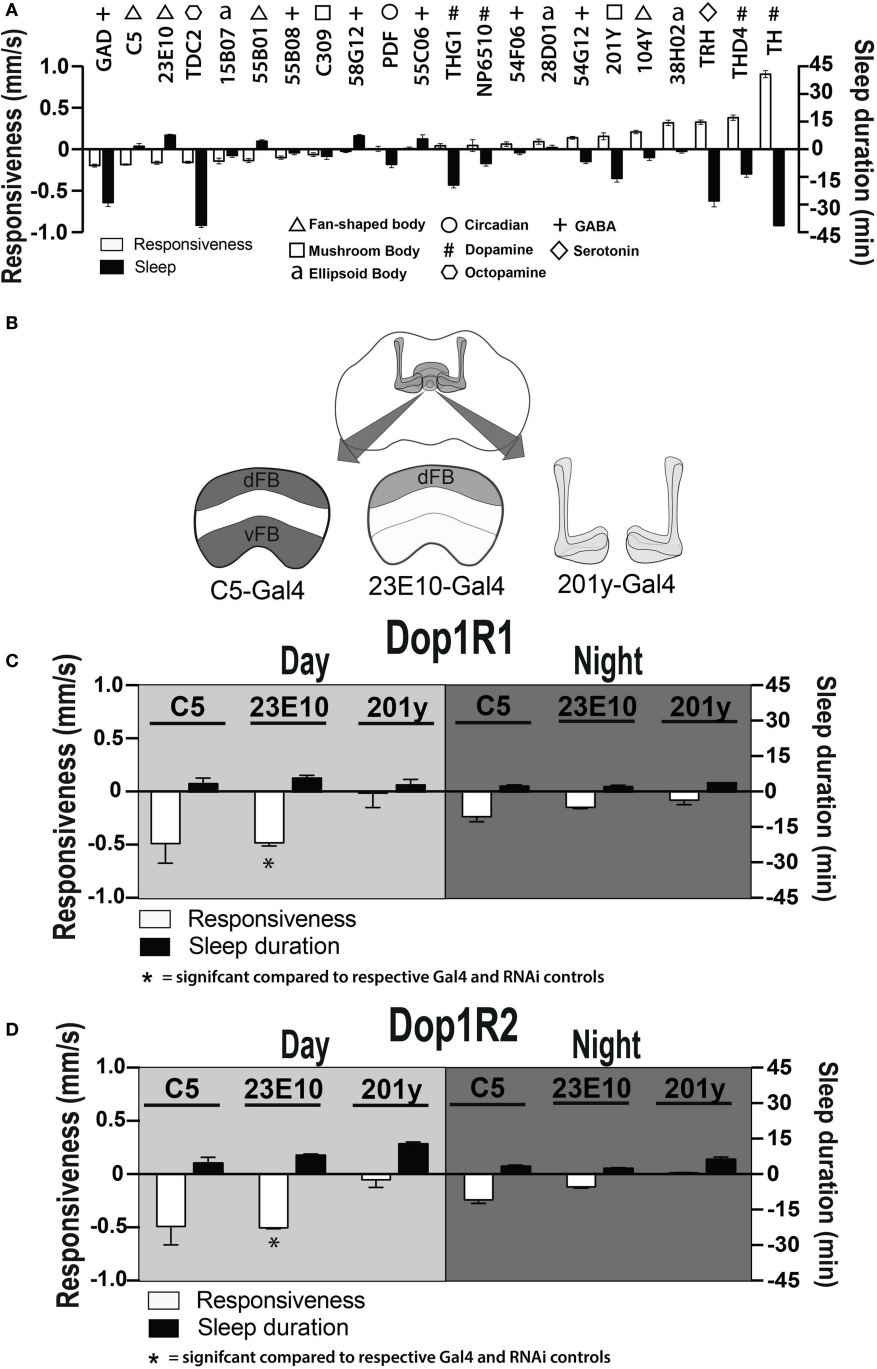
**Manipulating sleep-promoting neurons impairs behavioral responsiveness**. **(A)** 22 Gal4 circuits were activated with UAS-NachBac and resulting adult progeny were behaviorally characterized. Average nighttime responsiveness (white bars, mm/s ± SEM) or sleep duration (black bars, min ± SEM) for each strain is shown relative to the Gal4 genetic control. Different neuronal categories are indicated with symbols. *N* = 51 for all genotypes, including each respective Gal4 genetic control. **(B)** Schema of central brain regions associated with C5-Gal4, 23E10-Gal4, and 201y-Gal4 expression. dFB, dorsal fan-shaped body; vFB, ventral fan-shaped body. **(C)** Average daytime and nighttime responsiveness and sleep duration (±SEM) of treated C5-Gal4/UAS-Dop1R1; tubulin (tub)-Gal80TS animals, 23E10-Gal4/UAS-Dop1R1; tub-Gal80TS animals, and 201y-Gal4/UAS-Dop1R1; tub-Gal80TS animals (*N* = 56, 81, and 56, respectively), normalized to their corresponding Gal4 control (C5-Gal4/+; *N* = 108, 23E10-Gal4/+; *N* = 159, 201y-Gal4/+; *N* = 41), and compared to both Gal4 and RNAi (UAS-Dop1R1; tub-Gal80TS/+, *N* = 81, not shown) genetic controls. **(D)** Average daytime and nighttime responsiveness and sleep duration (±SEM) of treated C5-Gal4/UAS-Dop1R2; tub-Gal80TS animals, 23E10-Gal4/UAS-Dop1R2; tub-Gal80TS animals and 201y-Gal4/UAS-Dop1R2; tub-Gal80TS animals (*N* = 34, 66, and 30, respectively), normalized to their corresponding Gal4 control (C5-Gal4/+; *N* = 108, 23E10-Gal4/+; *N* = 159, 201y-Gal4/+; *N* = 41) and compared to both Gal4 and RNAi (UAS-Dop1R2; tub-Gal80TS/+, *N* = 32, not shown) genetic controls. *P < 0.05, by one-way ANOVA, adjusted for multiple comparisons by a *Post Hoc* Tukey’s test.

Dopamine has been found to act as an inhibitory neuromodulator in *Drosophila* when acting *via* D1 receptors (36, 37). Downregulation of D1 receptor function could thus cause persistently increased activity in specific neurons targeted by dopamine, thereby potentially explaining the altered responsiveness phenotypes that we have uncovered. To explore which postsynaptic circuits might be involved, we increased neuronal activity during development (38) in several circuits throughout the *Drosophila* brain and looked for circuits that decreased behavioral responsiveness in adults (Figure 7A, white bars), with a goal to uncover activated circuits that might mimic our developmental D1 knockdown effects (Figure 6B). We also measured sleep duration in these strains (Figure 7A, black bars). Six out of 22 activated circuits significantly decreased responsiveness to the mechanical stimulus (*P* < 0.001, *t*-test), notably GABA *via* Gad-Gal4 (39). That GABA activation decreases responsiveness is not surprising, since it is an inhibitory neurotransmitter, although the correlated decrease in sleep duration was unexpected (Figure 7A). Of the next five circuits that significantly decreased behavioral responsiveness, three of them drive expression of proteins in the dorsal fan-shaped body (dFB) of the central complex: C5-Gal4, GR23E10-Gal4, and GR55B01-Gal4 (Figure 7A, triangles). Neurons in the dFB have been described as sleep-promoting neurons (36, 40), and indeed constitutively activating two of these drivers (GR23E10-Gal4 and GR55B01-Gal4) also significantly increased sleep duration in adult flies (*P* < 0.001 and *P* < 0.01, respectively, by *t*-test). Interestingly, activating wake-promoting dopaminergic neurons (Th-Gal4) had exactly the opposite effect from the sleep-promoting neurons, by dramatically decreasing sleep and increasing responsiveness (*P* < 0.001, *t*-test) (Figure 7A, #). Activation of a DA sub-cluster that targets the dFB (THD4-Gal4) (24) had the same effect as Th-Gal4 (Figure 7A, #). Together, these results support the view that this sleep/wake circuit (i.e., dopaminergic input to the dFB) modulates behavioral responsiveness in addition to its predicted effects on sleep duration. These results suggest a postsynaptic locus for our original ontogenetic effects on behavioral responsiveness, namely in the sleep-promoting neurons of the dFB.

### Transient Dop1R1 Knockdown in dFB Neurons Recapitulates Behaviors Induced by Elevating Presynaptic Dopamine

We next asked whether transient D1 knockdown in dFB sleep-promoting neurons was sufficient to permanently alter behavioral responsiveness in adult animals. We used a broader sleep-promoting Gal4 driver [C5-Gal4 (35, 40, 41)] as well as a more restricted driver that expresses in less than 20 cells that innervate the dFB [GR23E10 (36, 40)] (Figure 7B). We transiently knocked down D1 receptors in these neurons by exposing pupae to an elevated temperature for 2 days (thereby transiently inactivating the Gal4 suppressor Gal80TS). When we tested treated flies as adults, we found that this localized D1 receptor knockdown (either Dop1R1 or Dop1R2) mimicked the effect seen in our original Th-Gal4/TrpA1 and D1-RNAi manipulations: flies displayed significantly decreased responsiveness to mechanical stimuli during the day, although this was significant only for Dop1R1 and Dop1R2 knockdown in the more restricted sleep-promoting driver, GR23E10 (Figures 7C,D, white bars) [day (*F*_(4, 23)_ = 6.866, Dop1R1, *P* = 0.0181; Dop1R2, *P* = 0.0306); night (*F*_(4, 23)_ = 2.864, Dop1R1, *P* = 0.1576, Dop1R2, *P* = 0.4936, Tukey’s)]. In contrast, when we performed the same manipulations in another group of neurons also associated with arousal and sleep, the mushroom bodies (MB) (42), we saw no such effect (201y, Figures 7C,D) [day (*F*_(4, 12)_ = 4.215, Dop1R1, *P* > 0.9999, Dop1R2, 0.9998, Tukey’s); night (*F*_(4, 12)_ = 4.210, Dop1R1, *P* > 0.9399, Dop1R2, 0.9993, Tukey’s)].

**Figure 8:**
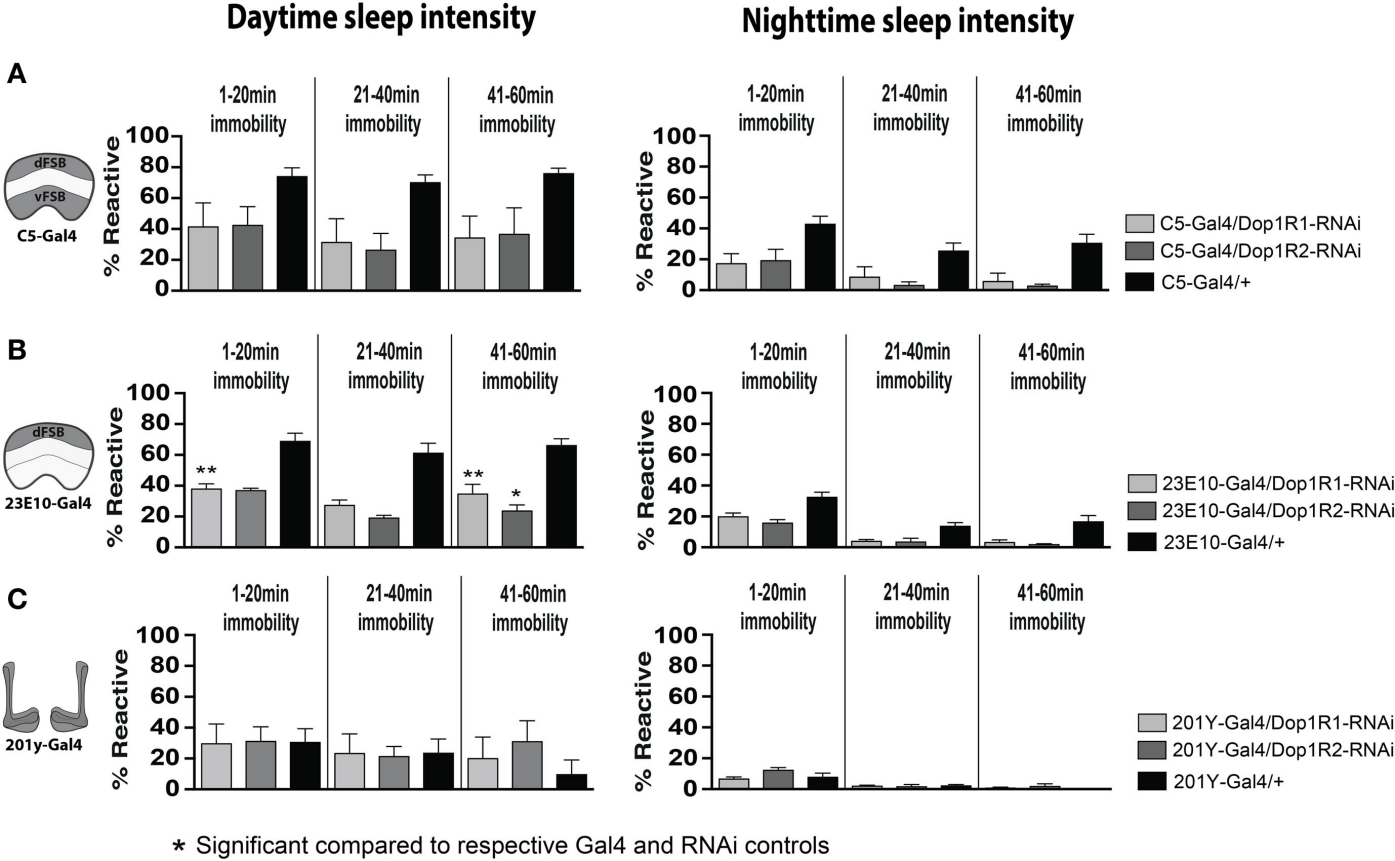
**Transient 23E10-Gal4 driven knockdown of D1 receptors during development increases daytime sleep intensity in adult flies**. **(A)** Daytime (left) and nighttime (right) sleep intensity (% reaction proportion ± SEM) of treated C5-Gal4/UAS-Dop1R1; tubulin (tub)-Gal80TS animals (light gray, *N* = 56) and C5-Gal4/UAS-Dop1R2; tub-Gal80TS (dark gray, *N* = 34) compared to C5-Gal4/+ (black, *N* = 108) and RNAi (UAS-Dop1R1; tub-Gal80TS/+, *N* = 81, not shown) genetic controls. **(B)** % reaction proportion (±SEM) of treated 23E10-Gal4/UAS-Dop1R1; tub-Gal80TS animals (light gray, *N* = 81), and 23E10-Gal4/UAS-Dop1R2; tub-Gal80TS (dark gray, *N* = 66) during the day (left) and night (right) compared to 23E10-Gal4/+ (black, *N* = 159). *P < 0.05, **P < 0.01, decreased% reaction proportion compared to both genetic controls, by one-way ANOVA, adjusted for multiple comparisons by a *Post Hoc* Tukey’s test. **(C)** % reaction proportion (±SEM) of treated 201y-Gal4/UAS-Dop1R1; tub-Gal80TS animals (white, *N* = 56) and 201y-Gal4/UAS-Dop1R2; tub-Gal80TS (dark gray, *N* = 30) compared to 201y-Gal4/+ (black, *N* = 41) and RNAi (UAS-Dop1R2; tub-Gal80TS/+, *N* = 32, not shown) genetic controls.

Since the dFB neurons have been intimately linked with sleep-homeostatic regulation (e.g., sleep duration) rather than behavioral responsiveness, we then examined whether transient D1 knockdown in these neurons (C5-Gal4, 23E10-Gal4) altered sleep duration in adult flies. Interestingly, sleep duration was not significantly (*P* > 0.05, Tukey’s) changed compared to genetic controls, although there was a trend to increased sleep (Figures 7C,D, black bars). Consistent with our other results, sleep intensity was increased during the day when D1 knockdown was restricted to the dFB (23E10: day, *F*_(14, 69)_ = 9.721; 1–20 min, Dop1R1, *P* < 0.01; 41–60 min, Dop1R1, *P* < 0.01, Dop1R2, *P* < 0.05) (Figures 8A,B), but not when restricted to the MB (Figure 8C). No significant effects for nighttime sleep intensity were found. This again shows that the developmental manipulation permanently alters behavioral responsiveness, even when restricted to D1 receptor function in a subset of sleep-promoting neurons. Indeed, throughout our study, behavioral responsiveness appears to be much more sensitive to developmental manipulations than sleep duration, whether as a consequence of transient DA activation or transient DA receptor knockdown.

## Discussion

In this study, we found that transient dopaminergic manipulations during brain development can have long-lasting effects on behavioral responsiveness in adult flies. We suggest that this model might be used to understand the development of cognitive disorders such as schizophrenia. The transient nature of our DA manipulation potentially mimics transient effects during gestation that might increase the likelihood of developing disorders of salience allocation later in life. There have been several other *Drosophila* studies examining fly behavior following various DA manipulations, and it is interesting to note that often the effects on behavior are quite subtle (10). For example, permanently abolishing DA synthesis in the fly nervous system impairs arousal but leaves several complex brain functions largely intact (43). We show here that a transient loss of balance in the DA system during development—a more likely scenario during human gestation—can be highly consequential for adult brain function.

We found that increased DA activity during development permanently decreases arousal in adult flies. This effect was evident across different sensory modalities, with treated flies responding less strongly to both mechanical and visual stimuli, compared to similarly treated genetic controls. In addition to being less responsive while awake, DA-manipulated flies also slept more deeply during both the day and night. This is likely to reflect generally decreased behavioral responsiveness levels, rather than any defects in sleep homeostasis. Consistent with this view, sleep duration was less easily perturbed in DA-manipulated animals. Closer examination of locomotion behavior in these animals also revealed some subtle defects: when exposed to moving gratings (a stimulus designed to evoke an optomotor response), treated animals paused more often. This observation in individual flies may explain why, in an earlier population-level study (13), we observed increased optomotor responses in flies that underwent this same developmental manipulation. Increased pausing in a population-level binary choice paradigm (44) might allow for greater integration of optomotor cues and thereby promote optomotor responsiveness. Importantly, the individual fly assays utilized in the current study better identify the defect resulting from the transient DA manipulation. This appears to primarily be decreased behavioral responsiveness across different sensory modalities, which is consistent with other studies showing that DA regulates general arousal in *Drosophila* (10, 43).

Remarkably, we were able to recapitulate the effects of the developmental DA manipulation on arousal by transiently knocking down D1 receptor levels during development. Although D2 receptor malfunction has been linked to hypolocomotion in a previous *Drosophila* study (33), more recent work has linked D1 receptors in the central fly brain to sleep and arousal phenotypes (24, 25, 34–36). Given this similarity in adult behavioral phenotypes, our results suggest that increased dopamine activity during development might lead to a compensatory downregulation of DA receptors. Either outcome (increased DA activity or decreased D1 expression) could therefore produce a persistent alteration in the ontogeny of DA systems. Our results are consistent with a recent study also showing downregulation of D1 receptors following constitutive upregulation of DA (31); our study shows that even transient downregulation of D1 during development can have persistent effects on behavior in adults. When we restricted the transient D1 knockdown to fewer neurons, we found that the dFB of the central complex forms a likely postsynaptic target for our original manipulation. It is unclear whether dFB neuroanatomy has been altered following the developmental DA manipulations, although in our previous study, we did not notice any gross morphological changes in this and other structures (13). This suggests that changes in the dFB might be primarily postsynaptic.

The dFB is an important structure in the fly brain for regulating arousal levels. A number of studies already suggest that this group of neurons is sleep promoting (24, 25, 35, 36, 40, 41, 45) and that DA modulates these neurons *via* D1 receptors (24, 25, 35, 36). However, the dFB neurons are also likely to be involved in regulating arousal more generally, as they also respond to visual stimuli in a state-dependent manner (46) and have been associated with visual learning (47, 48). It is interesting to note that developmental D1 knockdown in the dFB did not significantly impact sleep duration in adults. The robustness of the sleep duration readout is surprising, because both DA (which is wake promoting) and the dFB (which is sleep promoting) strongly affect sleep duration in opposite ways (as was evident when these circuits are constitutively activated, Figure 7A). That behavioral responsiveness is more strongly affected by developmental DA activation or D1 knockdown in the dFB neurons suggests that the affected sleep-promoting neurons also regulate arousal and that behavioral responsiveness is much more sensitive to a developmental dysregulation of these neurons, compared to sleep duration. Our results thus suggest that dFB neurons might be primarily regulating behavioral responsiveness, from which sleep duration may be a secondary consequence.

The dopamine ontogeny hypothesis has been proposed as an explanation for the development of psychotic symptoms in schizophrenia (3). Here, we have directly manipulated dopaminergic pre- and postsynaptic signaling during late pupal development in the fly. Our motivation for such experiments is to better understand how subtle, transient alterations in developmental dopamine circuitry can lead to persistent alterations in adult brain function. These experiments are consistent with the DA ontogeny hypothesis of schizophrenia where it is proposed that certain adverse environmental factors during brain development converge on vulnerable developing dopaminergic circuits to produce a brain in which dopamine signaling is persistently altered. The fly may prove an attractive model organism for future investigations of DA neurobiology that is susceptible to adverse environmental exposures. For example, recent work has shown that infection by *Wolbachia* bacteria alters monoamine levels in *Drosophila* (17). Other stressors such as starvation can also alter DA levels in the fly model (49). It will be interesting to see whether some environmental stressors might have a similar effect on dopamine ontogeny and adult behavior as our focused thermogenetic manipulations.

Our localization of DA signaling deficits in developing dFB neurons provides an extremely targeted anatomical focus for further testing of the dopamine ontogeny hypothesis, as we were able to recapitulate behavioral phenotypes by targeting our manipulations to as few as ~20 dFB neurons. Our study has also provided important new data further delineating the role of DA circuits in regulating different fly behaviors. Given the conserved role of dopamine in modulating arousal across species (10), our findings may inform our understanding of comparable mechanisms in humans. Future genetic and pharmacological studies could be designed to target the DA-dFB circuit, we have uncovered in an attempt to correct such aberrant developmental processes, thus potentially informing future preventative therapies in schizophrenia.

## Author Contributions

LF and AP designed and performed experiments, analyzed data, and wrote the paper. CR and MT contributed and analyzed data. LK designed experiments and analyzed data. DE and BvS conceived the study, designed experiments, analyzed data, and wrote the paper.

## Conflict of Interest Statement

The authors declare that the research was conducted in the absence of any commercial or financial relationships that could be construed as a potential conflict of interest.
